# Comparative genomics of clinical strains of *Pseudomonas aeruginosa* strains isolated from different geographic sites

**DOI:** 10.1038/s41598-018-34020-7

**Published:** 2018-10-23

**Authors:** Dinesh Subedi, Ajay Kumar Vijay, Gurjeet Singh Kohli, Scott A. Rice, Mark Willcox

**Affiliations:** 1 School of Optometry and Vision Science, University of New South Wales, Sydney, Australia; 2 Singapore Centre for Environmental Life Sciences Engineering, Singapore, Singapore; 3 The School of Biological Sciences, Nanyang Technological University, Singapore, Singapore; 4 The ithree Institute, The University of Technology Sydney, Sydney, NSW Australia

## Abstract

The large and complex genome of *Pseudomonas aeruginosa*, which consists of significant portions (up to 20%) of transferable genetic elements contributes to the rapid development of antibiotic resistance. The whole genome sequences of 22 strains isolated from eye and cystic fibrosis patients in Australia and India between 1992 and 2007 were used to compare genomic divergence and phylogenetic relationships as well as genes for antibiotic resistance and virulence factors. Analysis of the pangenome indicated a large variation in the size of accessory genome amongst 22 stains and the size of the accessory genome correlated with number of genomic islands, insertion sequences and prophages. The strains were diverse in terms of sequence type and dissimilar to that of global epidemic *P*. *aeruginosa* clones. Of the eye isolates, 62% clustered together within a single lineage. Indian eye isolates possessed genes associated with resistance to aminoglycoside, beta-lactams, sulphonamide, quaternary ammonium compounds, tetracycline, trimethoprims and chloramphenicols. These genes were, however, absent in Australian isolates regardless of source. Overall, our results provide valuable information for understanding the genomic diversity of *P*. *aeruginosa* isolated from two different infection types and countries.

## Introduction

The diverse and dynamic genetic composition of *Pseudomonas aeruginosa* enables this Gram-negative bacterium to colonise various environments, including humans where it can cause opportunistic infections^1,2^. *P*. *aeruginosa* is particularly associated with infections that are caused due to impaired anatomical structures or a weakened immune system. Such infections include microbial keratitis (MK), ventilator-associated pneumonia, wound infections, and respiratory infections in patients suffering from cystic fibrosis (CF)^3–5^. Several reports have shown that the prevalence of such infections by multidrug-resistant (MDR) strains is increasing rapidly worldwide^6–9^, which makes this bacterium difficult to treat and hence there is a high risk of mortality associated with infection by *P*. *aeruginosa*^10^. This pathogen has an exceptional capacity to develop resistance to antibiotics by the selection for genomic mutations and by exchange of transferable resistance determinants^11^. Knowledge of the genomic diversity of *P*. *aeruginosa* will help to understand differences in pathogenesis between strains and the mechanism of antibiotic resistance, which is important for controlling infections.

The genome size of *P*. *aeruginosa* varies greatly, ranging from 5.5 to 7 Mbp^12,13^. Such variation arises due to the presence of a large accessory genome. Accessory genomes are strain specific blocks of DNA and can occupy up to 20% of the whole genome^14^. They are composed of horizontally transferable elements which include prophages, transposons, insertion sequences (IS), genomic islands (GI) and plasmids^15^. Accessory genomes are important for carrying virulence and acquired antibiotic resistance genes. The lateral transfer of those genes between strains contributes to the development of MDR virulent strains^16^. Furthermore, mutational changes of chromosomal genes can also contribute to virulence and antibiotic resistance^16,17^. Therefore, unraveling the genetic content of *P*. *aeruginosa* helps to understand the gene modifications that are associated with more pathogenic and more resistant strains. Several studies have reported a comparison between genomes of *P*. *aeruginosa* in different infections at various points of time during infections^16,18–21^. However, most of those studies have centered around CF isolates and there is very limited comparative genomics of ocular isolates of *P*. *aeruginosa*.

This study aims to compare the genomic diversity between *P*. *aeruginosa* strains from MK and CF isolated in Australia and India. There are previous reports of genomic characterisation of Indian ocular isolates of *P*. *aeruginosa*^22–24^. A genotypic study of eye isolates of *P*. *aeruginosa* has shown that keratitis isolates from the UK are highly related^25^. However, information on genomic comparison amongst contemporary isolates of *P*. *aeruginosa* from eye infections in different geographical locations is still missing. This study focussed on 13 MK strains, which were isolated in India and Australia and nine strains from CF cases which were isolated in Australia. The whole genomes of all 22 strains were sequenced and a comparative genomic analysis was conducted to identify genomic divergence, evolutionary relationships, antibiotic resistance properties and virulence factors.

## Results and Discussion

### General features of genomes

A *de novo* assembly of the genomes of 22 *P*. *aeruginosa* strains generated a number of contigs from 56 in PA175 to 241 in PA37 (Median = 79). Like other published complete genomes of *P*. *aeruginosa*^1,19,26,27^, a mean C + G content of 66.4% and size of 6.1 to 7.1 Mbp was observed in the draft genomes. The genomic size varied widely between strains showing up to 900 kbp more DNA than PAO1, which was taken as the reference strain in this study. Similarly, the number of coding sequences (CDS), which were determined based on Prokka annotation pipeline, ranged from 5584 (in PA92) to 6645 (in PA37). Amongst 82 complete genomes of *P*. *aeruginosa* listed in the Pseudomonas genome database (PGDB)^28^ (accessed on 12/03/2018), PA92 has the lowest and PA37 has the second highest number of CDS. Wide variations in the tRNA copy numbers (65–73) per strain observed here is probably due to use of incomplete draft genome. In addition, different number of tRNAs in the same genome was observed when annotated using different pipelines. Table 1 shows the general features of the genomes.

**Table 1:** General features of the genomes of *P*. *aeruginosa* strains.

	Strains	Sequence type^#^	No. of contigs	Length (bp)	GC (%)	CDS	tRNA	Accessory genes^##^
Eye/India	PA31	308	137	7100578	66.02	6619	69	1709
	PA32	308	155	7101589	66.01	6611	69	1701
	PA33	308	166	7092617	66.02	6609	69	1699
	PA34	1284	130	6885314	65.95	6326	66	1416
	PA35	308	156	7094960	66.02	6611	69	1701
	PA37	308	241	7154765	65.94	6645	69	1735
	PA82	1027	64	6387501	66.51	5810	65	900
								Average number of accessory genes = 1552
Eye/Australia	PA17	New	60	6360710	66.45	5825	72	915
	PA40	New	109	6284606	66.44	5700	69	790
	PA149	New	59	6314825	66.46	5745	68	835
	PA157	386	56	6249622	66.53	5708	68	798
	PA171	471	60	6339342	66.49	5812	69	902
	PA175	309	62	6757641	66.2	6181	68	1271
								Average number of accessory genes = 919
CF/Australia	PA55	549	77	6235554	66.57	5668	67	758
	PA57	New	73	6333117	66.48	5792	68	882
	PA59	New^†^	78	6289887	66.55	5767	68	857
	PA64	775	87	6264428	66.55	5713	65	803
	PA66	New^†^	93	6337310	66.51	5828	68	918
	PA86	New^†^	76	6170893	66.46	5685	68	775
	PA92	775	81	6144573	66.59	5584	65	674
	PA100	483	83	6310616	66.5	5732	66	822
	PA102	1717	62	6245474	66.55	5710	69	800
								Average number of accessory genes = 810
	PAO1^*^	549	1	6264404	66.6	5671	73	761

^#^Sequence types were determined by the multi locus sequence typing database. The sequence types not listed in the MLST database have been deemed as new.

^##^Accessory genes were determined by subtracting number of core genes (4910) from total number of CDS. ^†^Same MLST allelic profile. *Reference strain.

A total of 9786 orthologs were detected in all 22 strains and the reference strain PAO1. As the pan-genome represents the cumulative genetic information within a set of bacterial genomes, its size increases with the number and diversity of strains used for evaluation. A study that included 17 *P*. *aeruginosa* reference strains from diverse sources has found 9344 orthologs in the pangenome^29^, which is comparable to the results observed here. The higher number of genes in the pan-genome in our study may be the result of the diverse nature of the studied strains. Out of the 9786 pan genes, 4910 genes were common in at least 99% of strains and this represents the core genome for the strains in the current study. Prior studies have reported core genomes of 5316^30^, 5233^29^, 5021^31^, and 4934^14^ in different *P*. *aeruginosa* strains. Although the other studies used smaller sets (5 to 17) of genomes, the results are broadly comparable. Many factors may be responsible for the smaller core genomes in the current study including a larger population of genomes used for alignment, use of incomplete draft genomes, the diverse nature of the study populations (ocular and lung; Australian and Indian) and a strict definition of the core genome (≥99% similarity in each strain). For example, pan-genome analysis of the same set of genomes of the current study but excluding PA57 and using ≥95% similarity resulted in 5287 core genes.

**Figure 1:**
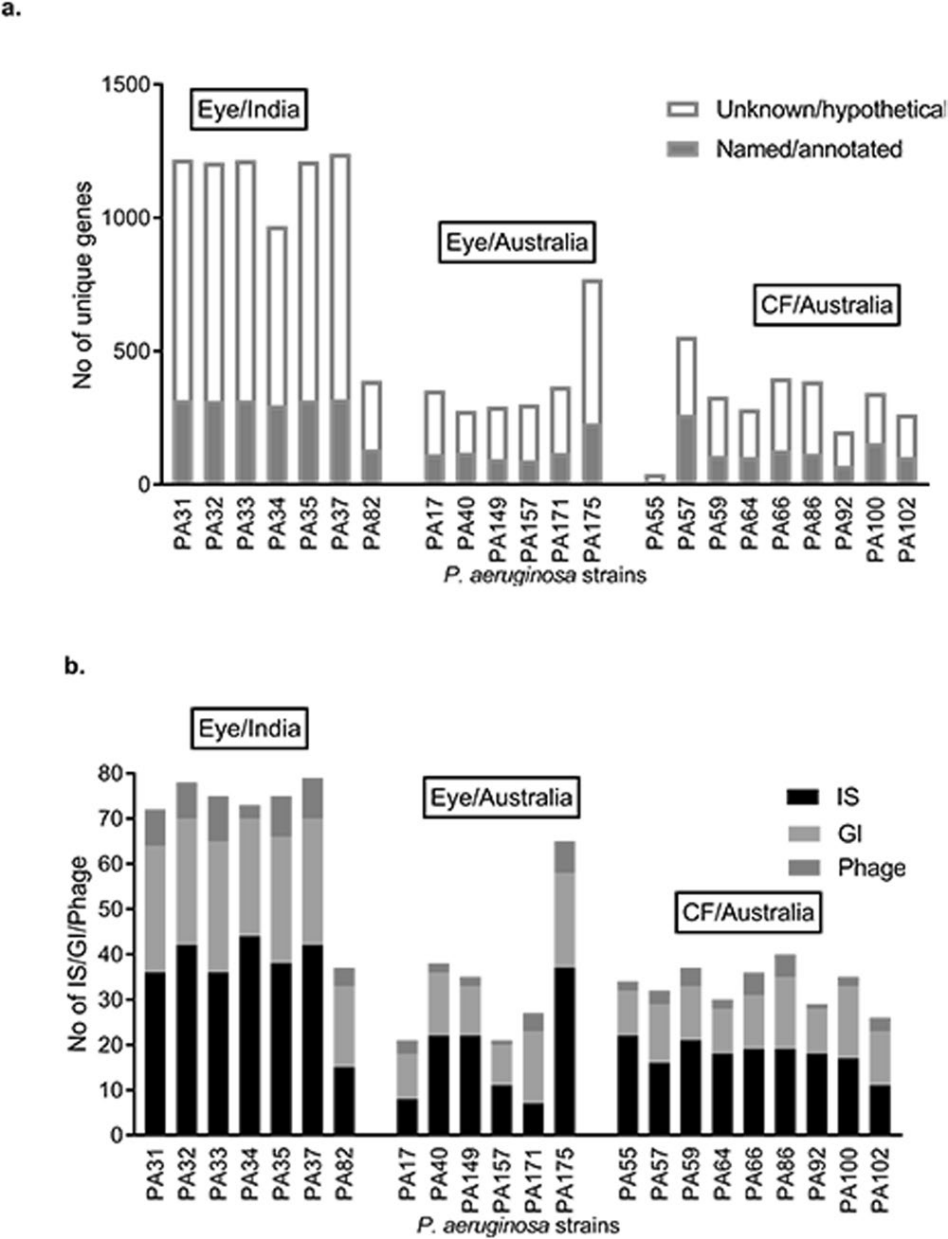
Composition of accessory genomes. (**a**) Distribution of unique genes. (**b**) Distribution of predicted no of insertion sequences (IS), genomic islands (GI) and phages.

In addition to the large core genome, *P*. *aeruginosa* has accessory genomes that are not common in all strains^15^. The accessory genome can comprise of up to 20% of the total genome, and the majority of genes in this accessory genome are acquired horizontally. These genes include phages, transposons, IS and GI^14^. In the current study, the accessory genes were identified by subtracting the core genes (4910) from the total number of CDS. The frequency of accessory genes was 12% to 26%, which is more than the previously reported size of accessory genome^29,32^. However, the use of draft genomes may overestimate the number of accessory genes because of the presence of genomic repeats or transposable elements that may interrupt assembly and give an apparently larger genome than this actually present^33^. Accessory genomes may carry genes that help strains to persist in environments that may be unsuitable for others^30^. Like many other bacteria, the accessory genomes of *P*. *aeruginosa* encompasses genes related to virulence and antibiotic resistance^34,35^. The presence of a higher number of accessory genes in the set of ocular isolates indicates that eye strains may have acquired many genes to make this opportunistic species suitable to grow in the ocular environment. Furthermore, we examined the number of unique genes amongst accessory genes and found that the functions of the majority of the unique genes are unknown (Fig. 1a).

The genomes were examined for the presence of insertion sequences (IS), genomic islands (GI) and prophages, which are the main elements of an accessory genome^15^. Contigs of draft genomes were reordered with reference to PAO1 and the ordered contigs were joined together and made into a single FASTA file before examining databases. The results show that the average predicated number of GI was 26 (range 29–18) in Indian eye isolates, which was greater than that of Australian isolates (average 13) irrespective of source. Similarly, the average predicted number of IS and phages were higher in Indian eye isolates (Fig. 1b). Twenty (PA157) to 75 (PA33) total accessory elements were observed in all draft genomes. In contrast, a study has noted 38 to 53 accessory elements that are integrated into 89 potential genomic loci (region of genomic plasticity) in the complete genome of several *P*. *aeruginosa* strains^14^. Complete genomes are required to ascertain the actual number of genes in accessory genome. Nevertheless, the predicted number of IS, GI and phages was well correlated with the size of the accessory genome indicating that they contribute to the genomic diversity as highlighted in other studies^15,35,36^.

From the pangenome and MLST analysis (below), five Indian strains, isolated from different patients with different histories, were found to be clonal and showed at least 99.98% sequence similarity with each other in MUMmer3^37^ whole genome alignment. To avoid the overestimation of the accessory genome due to the dominance of a single clone, we obtained the nucleotide sequence of five additional Indian eye isolates from public databases^22–24^ and reran the pan genome analysis. The relative size of the accessory genome to PAO1 was examined (Supplementary Fig. S1). The results tend to show that the eye isolates have larger accessory genome than CF isolates. However, due to limited number of clonally diverse strains of Indian origin, further research on larger datasets is required to confirm this.

Based upon MLST analysis, 16 distinct sequence types (STs) were found, with seven of these constituting new types. The ST was assigned to each strain according to the matched number in the MLST database^38^. Any strain that did not match with the existing database was deemed to have a new ST. Five Indian ocular isolates (out of seven) belonged to ST 308, two Australian CF isolates corresponded to ST 775 and three Australian CF strains had identical allelic profiles but did not match with any existing ST in the MLST database (all MLST profiles are shown in Supplementary Table S4). The remaining 13 STs were unique, with only a single representative (Table 1). Our results show that these strains belong to a diverse range of STs and are not similar to previously described clinical epidemic isolates^39,40^. Five strains with ST 308, collected from keratitis patients from the same centre in India, indicate the strains were potentially acquired from the same source where these strains may persist. The most common genotype observed in this study, ST 308, was also reported in MDR hospital strains in France^39^. Although the MLST database does not contain all *P*. *aeruginosa* strains, our observations show the diverse nature of the strains, which were not related to so-called world epidemic STs (ST 235, ST 111, ST 175, ST 395)^39^. This result also contradicts the previous finding that some keratitis isolates were clonally related with ST 235 CF strains^41^.

### Phylogenetics

A total of 82 complete genomes of *P*. *aeruginosa* including PAO1 were downloaded from the NCBI database and used to compare the phylogenetic diversity of 22 strains from the current study. These 82 strains were listed in PGDB^28^ as a complete genome and could represent a global *P*. *aeruginosa* collection. Core genome alignment was generated using Parsnp of the Harvest Suite with PAO1 as the reference. The alignment was then used to construct a tree following previously described methods, with *P*. *aeruginosa* PA7, a taxonomic outliner^27^, as an outgroup. A multi-sample variant call file was generated from the core genome alignment and SNPs present in all strains were examined (Supplementary Tables S1 and S2). In total 284,252 SNP sites were identified amongst 104 isolates.

**Figure 2:**
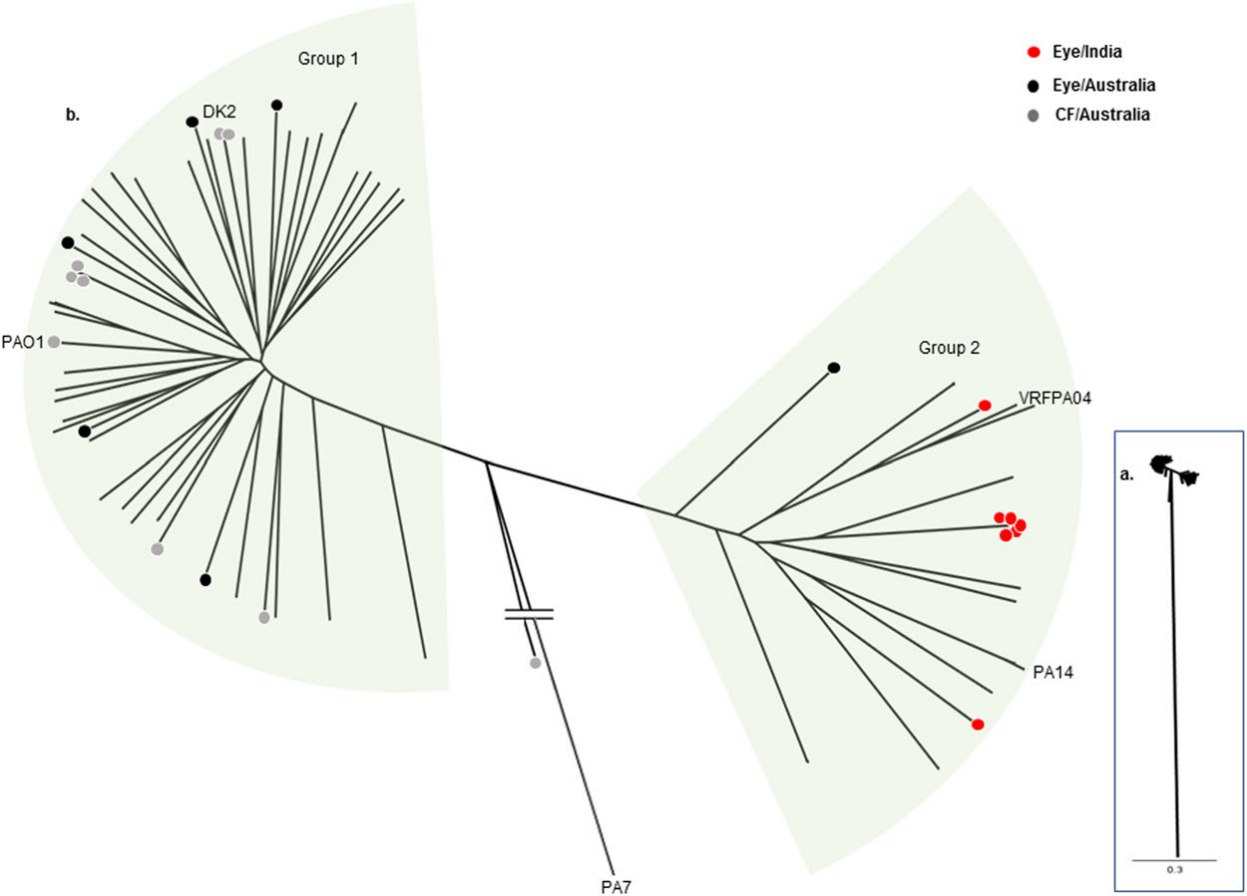
Phylogenetic analysis of *Pseudomonas aeruginosa* isolates. Maximum likelihood phylogenetic tree built with core genome SNPs based on mapping to the PAO1 excluding SNPs identified in regions that had arisen by recombination. (**a**) The original tree where the scale bars represent the number of substitutions per site. (**b**) Magnified tree showing branches and groups. Strains used in this study are indicated by distinct colour circles. Relative positions of few reference strains are shown, which are *P*. *aeruginosa* **VRFPA04**, *P*. *aeruginosa* UCBPP-**PA14**, *P*. *aeruginosa* **PAO1**, and *P*. *aeruginosa* **DK2**.

All strains, except PA57, were clustered into two groups (Fig. 2). This is in agreement with several studies which have also shown that *P*. *aeruginosa* strains from various sources tend to cluster into two major groups^42–44^, with group 1 being larger, and which contains the most widely studied stains PAO1^1^ and some notable CF strains such as DK2 and LESB58^45,46^. Group 2 tends to be smaller and includes the well known virulent strain PA14^13^ and an Indian ocular isolate VRFPA04, a virulent MDR strain^24^. All seven Indian and one Australian eye isolates were clustered into three sub-lineages within the group 2. A typing-based population structure analysis has also unveiled that keratitis *P*. *aeruginosa* strains are closely related^25^. Furthermore, this supports the finding of the previous study that human *P*. *aeruginosa* are less diverse than isolates from the environment^47^. Similarly, all the CF strains and five Australian eye strains were of group 1 (See Supplementary Table S1 for phylogeny group classification of each strains and associated core genome SNPs). Amongst the CF isolates, continuous mutations have been shown to be an evolutionary process that may make a strain more pathogenic so that they rapidly transfer between humans^16,21^. However, previous studies have not focussed on ocular isolates. Our analysis showed that more than 60% of eye isolates clustered together in a single group, which is in aggrement with previous findings that 71% of MK isolates of *P*. *aeruginosa* from the UK clustered together in the same group^48^. Further studies should focus on the evolutionary changes in ocular isolates of *P*. *aeruginosa* over a prescribed period of time. A CF strain PA57 was in a separate cluster and did not show similarity with other strains. This strain could be another taxonomic outlier of the *P*. *aeruginosa* (group 3)^44^.

### Antibiotic resistance gene profiles

**Figure 3:**
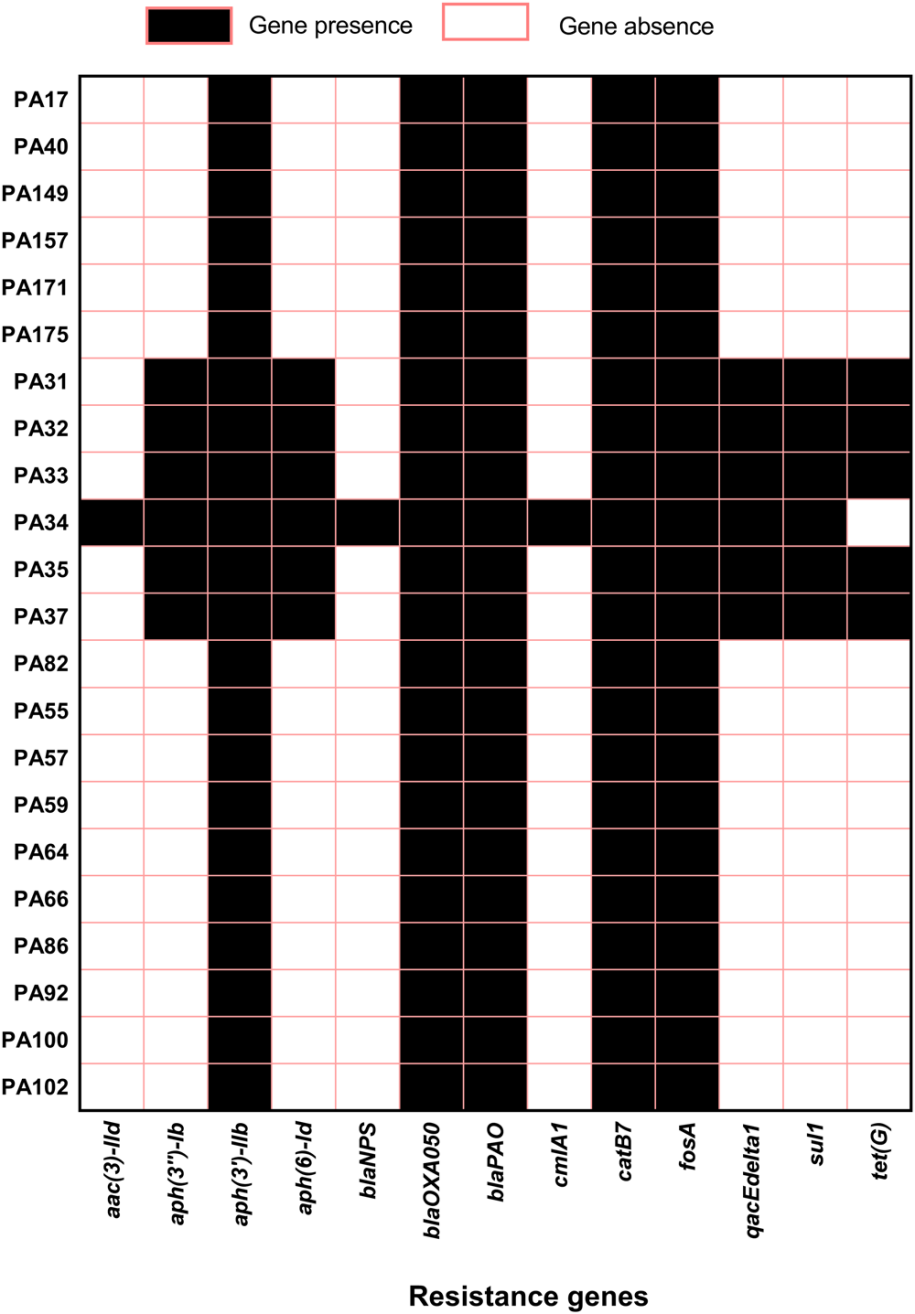
The presence and absence of resistance genes as detected by Resfinder database. Associated resistance: Beta lactams: - *bla*_OXA-50_, *bla*_PAO,_, *bla*_NPS-1_ Aminoglycosides: - *aph*(*3*′)*-IIb*, *aph*(*6*)*-Id*, *aph*(*3*″)*-Ib*, *aac*(*3*)*-IId* Fosfomycin: *fosA* Sulphonamide: *sul1* Chroramphenicol: *cmlA1*, *catB7* Tetracycline: *tet*(G). Quaternary ammonium compounds: *qacEdelta1*.

Horizontally acquired resistance genes were examined using the assembled contigs in the ResFinder database. Altogether, 13 distinct types of acquired resistance genes were detected in this study (Fig. 3). In common with other *P*. *aeruginosa* strains^24^, two beta-lactams (*bla*_OXA-50_, *bla*_PAO_) and one each for aminoglycoside (*aph*(*3*′)*-IIb*) fosfomycin (*fosA*) and chloramphenicol (*catB7*) resistance genes were present in all studied strains. Furthermore, six out of 22 strains had acquired additional resistance genes. Interestingly, all six strains were Indian eye isolates and possessed two aminoglycoside resistance genes (*aph*(*3*″)*-Ib* and *aph*(*6*)*-Id*), one sulphonamide resistance gene (*sul1*) and one quaternary ammonium compound resistance gene (*qacEdelta1*). The tetracycline efflux protein transporter gene *tet*(G) was detected in five Indian eye isolates, all of them are ST 308. An Indian eye strain PA34 possessed four unique resistance genes; *bla*NPS-1, *aac*(*3*)*-IIb*, *dfrA15* and *cmlA1* that can confer resistance to beta-lactams, aminoglycosides, trimethoprims and chloramphenicols, respectively. As horizontally acquired resistance genes may be associated with integrons, we analysed all of the draft sequences for the presence of integrons using Integron Finder version 1.5.1^49^. Although *sul1* and *qacEdelta1* are indicative of class I integrons, only strain PA34 possessed a class 1 integron, in agreement with a recent publication^50^. The acquired resistance genes detected were comparable to previous observations for an Indian eye isolate of *P*. *aeruginosa*^24^. As all Indian isolates of the current study were from the same centre in India, it is possible that there was antibiotic selection pressure that led to the selection for strains that had acquired such resistance genes from the environment. The absence of such genes in Australian isolates indicates that the antibiotic selection pressure may be different between Australia and India or that the genes associated with resistance are not readily accessible to *P*. *aeruginosa* in their local Australian environment. Furthermore, isolates from India were more likely to carry more resistance genes than Australian isolates, potentially reflecting the relatively unregulated use of antibiotics in India compared to Australia^51^. Antibiotic susceptivity tests also shows that Indian eye isolates were resistance to gentamicin and at least one fluoroquinolone. Resistance to aminoglycoside and fluoroquinolone is however, low in Australian isolates (Table 2).

**Table 2:** Antibiotic susceptibility profile of *P*. *aeruginosa* strains.

Strains		Antibiotics									
		Gentamicin	Ciprofloxacin	Levofloxacin	Moxifloxacin	Ceftazimidin	Cefepime	Imipenem	Ticarcillin	Aztronam	Polymyxin B
Eye/India	PA31	**R**	**R**	**R**	**R**	I	I	I	I	I	I
	PA32	**R**	**R**	**R**	**R**	I	S	I	I	S	I
	PA33	**R**	**R**	**R**	**R**	I	I	I	I	S	S
	PA34	**R**	I	S	**R**	S	I	**R**	I	I	S
	PA35	**R**	**R**	**R**	**R**	I	I	I	I	S	S
	PA37	**R**	**R**	**R**	**R**	I	S	I	I	S	S
	PA82	I	**R**	I	S	**R**	**R**	S	I	S	**R**
Eye/Australia	PA17	S	I	S	**R**	S	S	S	I	I	I
	PA40	S	**R**	S	S	S	S	I	I	S	S
	PA149	S	S	S	S	I	S	S	S	S	S
	PA157	S	S	S	S	I	S	S	I	S	S
	PA171	S	S	S	S	I	S	I	I	S	S
	PA175	S	I	S	S	I	S	I	I	S	S
CF/Australia	PA55	**R**	S	S	S	S	S	I	I	S	S
	PA57	**R**	S	S	S	S	S	S	I	S	S
	PA59	**R**	S	S	S	S	S	S	I	S	S
	PA64	I	**R**	**R**	**R**	S	**R**	S	I	S	S
	PA66	**R**	I	I	**R**	S	**R**	S	I	S	S
	PA86	S	S	S	S	I	S	S	I	S	S
	PA92	I	I	I	**R**	I	**R**	S	S	I	S
	PA100	I	I	S	**R**	I	S	S	S	S	S
	PA102	**R**	I	I	**R**	I	S	S	S	S	S

On the basis of searches in the literature and online databases (Comprehensive Antibiotic Resistance Database (CARD), https://card.mcmaster.ca/home and the Pseudomonas genome database, http://www.pseudomonas.com), a set of 73 genes, which were related to antibiotic and disinfectant resistance in *P*. *aeruginosa*, were selected to examine variations in these genes between strains. Only high-quality, non-synonymous SNPs and indels were used for interpretation (Table 3). No insertions or deletions were detected in any of the strains. In terms of the number of SNPs and strains types, all Indian eye isolates and one Australian eye isolate (PA175) had relatively more variations (total SNPs >125) in the set of resistance genes than other strains. However, the CF strain PA57 had an exceptionally high number of SNPs in its resistome. Another CF strain, PA55, did not show any variations in its resistome. In terms of the total SNPs in resistance genes, the least number of variations (≤5 SNPs) were found in five efflux pump-related genes (*oprM* (5) *cycB*(1) *mexF* (4) *nalD*(5) and *nfxB* (2)), three target alternation genes (*gyrB* (5) *tufA*(2) *tufB* (0)) and one inactivation gene *fosA* (3); these are highly conserved genes in *P*. *aeruginosa*.

**Table 3:** Non-synonymous SNPs detected in the 73 genes related to antibiotic resistance in the 22 isolates studied using PAO1 as the reference genome.

Gene locus	Gene name	Mechanism	*P. aeruginosa* strains/number of SNPs																						
			PA31	PA32	PA33	PA34	PA35	PA37	PA82	PA17	PA40	PA149	PA157	PA171	PA175	PA55	PA57	PA59	PA64	PA66	PA86	PA92	PA100	PA102	Total SNPs
PA0156	*triA*	Antibiotic efflux	4	5	5	1	6	5	4	1		1			3		1							1	37
PA0157	*triB*				1				1	1		2	1				1	1	1	1	1	1	1	1	14
PA0158	*triC*		2	2	2	1	2	2	1			1			1		3							1	18
PA0424	*mexR*		2	1	1	2	1	1	1						1		2								12
PA0425	*mexA*										1		1		1			1		1	1		1		7
PA0426	*mexB*		1	1	2		1	4									2		1			1			13
PA0427	*oprM*											1	1					1		1	1				5
PA1236	*farB*		1	1	1	1	1	1	1	1			1						1			1			11
PA1282	*lrfA*		6	6	9	4	6	8	6	8	5	5	6	4	4		7	3	4	3	3	4	3	4	108
PA1316	*lrfA*		2	2	2	1	2	2	3	3	2	1	3	2	3		3	2	2	2	2	2	2	4	47
PA1435	*mexM*		4	4	4	5	4	4	3	4	6	5	6	6	5		8	5	5	5	5	5	4	5	102
PA1436	*mdtC*		2	2	2	1	2	2	2	3	2		3	2	2			2	2	2	2	2	2	4	41
PA1754	*cysB*																						1		1
PA2018	*mexY*		5	5	5	5	5	5	3	1	1	1	4	1	3		3	1	1	1	1	1	1	2	55
PA2019	*mexX*		4	4	4	4	4	4	5	3	4	5	3	3	3		6	3	4	3	3	4	3	4	80
PA2389	*macA*		2	1	1	1	1	1	1		1		1	1	1		1	1		1	1		1	1	18
PA2390	*macB*		1	1	1	3	1	1	2	1	3	2	3	1	1		3	1	2	1	1	2	1	3	35
PA2391	*opmQ*		6	5	6	4	6	6	4	1	3	2	1	2	4		5	4	5	4	4	5	3	1	81
PA2491	*mexS*		2	2	2	1	2	2	2	1	1	1	1	1	1		4	2	2	2	2	1	1	1	34
PA2493	*mexE*					1				1				1	3		2	1	2	1	1	1			14
PA2494	*mexF*					1			1								2								4
PA2495	*oprN*		1	1	1	2	1	1	1		1	1			1		2		1			1			15
PA2525	*adeC*					3						5	1		1		2	2			2		3	1	20
PA2526	*muxC*										1			1			1	1		1	1				6
PA2527	*muxB*					1					2				1										4
PA2837	*opmA*		3	3	3	4	3	3	5		1	1		3	4		6	2	1	2	2	1	1	2	50
PA3019	*taeA*		1	1	1	3	1	1	1	1	1	2	2	2	2		4	1	1	1	1	1	3	1	32
PA3137	*farB*		1	1	1	1	1	1		1	2		2	2	2		2	1	1	1	1	1	1	1	24
PA3521	*opmE*		3	3	3	2	3	3	2	3	5	6	4	2	3		11	3	5	3	3	5	3	2	77
PA3522	*mexQ*		4	4	4	4	4	4	6	4	5	2	2	3	3		3	4	6	4	4	5	2	3	80
PA3523	*mexP*		2	2	2	1	2	2	2	3	2		2	1	2		3		3			3	2	2	36
PA3574	*nalD*									1				1					3						5
PA3676	*mexK*		1	1	1	2	1	1	5	1	2		2	2	3		6	4	2	4	4	2	2	2	48
PA3677	*mexJ*		2	2	2	2	2	2	2			1			2		3	3	3	3	3	3	1		36
PA3678	*mexL*		1	1	1	1	1	1	1	1		1	1		1		2	2	1	2	2	1			21
PA3894	*adeC*					1			1			1	1	1			2	2	1	2	2	1	1		16
PA4205	*mexG*		1	1	1		1	1	1								3								9
PA4206	*mexH*		1	1	1	2	1	1							1		1								9
PA4207	*mexI*		1	1	1	1	1	1	1	1			1	1	1			1	1	1	1	1			16
PA4208	*opmD*		3	3	3	3	3	3	2	5	1	1			4		6							1	38
PA4374	*mexV*		2	2	2	4	2	2	3		2	1	1	2	2		4	1	2	1	1	2	1	3	40
PA4375	*mexW*		2	2	2	1	2	2	3	3	1	2	3	1	1		2	1	1	1	1	1			32
PA4595	*yjjk*					1				1		2		1	2		1	3	1	2	1	1		1	17
PA4597	*oprJ*									2	2	2	2				9	3	3	5	3	3	3		37
PA4598	*mexD*		2	2	2	2	2	2	2	3	2	2	1	3	2		9	2	1	2	2	1	2	3	49
PA4599	*mexC*		7	8	8	3	8	8	6	4	1	4		1	5		9	1		1	1		4	4	83
PA4600	*nfxB*												1				1								2
PA4974	*opmH*		2	1	1	1	5	5			1			2	1		5	1	5	1	1	3	4		39
PA4990	*emrE*		1	1	1		1	2				1							1			1			9
PA4997	*msbA*		2	3	3	2	2	3	4						4		3		1			1		1	29
PA5158	*adeC*		3	3	3		3	3	3	2	2	1	2		3		5	3	3	3		3	2	1	48
PA5160	*farB*		4	3	3	2	4	3	5	3	3	3	3	3	3		3	3	4	3		3	4	3	65
PA5518	*rosB*		3	3	3	2	3	3	3					1	1		1	1	1	1		1	1	1	29
PA0706	*catB7*	Antibiotic inactivation	4	4	4	5	4	4	3	3	2	2	2	3	4		1	2	4	2	2	4	3	3	65
PA1129	*fosA*										1						2								3
PA4109	*ampR*		2	2	2	3	2	2	1					2	2		3								21
PA4110	*ampC*		5	5	5	3	5	5	6	1	2	2	2	2	5		12	1	1	1	1	1	2	2	69
PA4119	*Aph*(*3*′)*-IIb*		2	2	2	3	2	2	2	1	1	1	1	4			3		1			1	1	2	31
PA5514	*OXA-50*		1	2		1	3	2	5	2	3		2	3	3		4	1	3	1		3		4	43
PA0004	*gyrB*	Antibiotic target alternation																		1	3	1			5
PA0903	*alaS*		1	1	1	1	1	1	1		1							1		1	1		2		13
PA1972	*pmrC*		3	3	3	3	3	3	1	1	1	4	1	1	2		3	2	3	2	2	3	4	1	49
PA3002	*mfd*		1	2	2	3	2	2	1	1	1		2		1		2	1	2	1	1	2			27
PA3168	*gyrA*		1	1	1		1	1							2		2		1					1	11
PA3946	*rosC*		6	8	8	3	7	6	1	1	1	1	2	2				1	2	1	1	2	2	1	56
PA4265	*tufA*		1					1																	2
PA4277	*tufB*																								0
PA4560	*ileS*		2	2	2	2	2	2	2	1	4	2	1	1	2		4	2	4	2	2	4	3	1	47
PA4964	*parC*		2	2	2		2	2			1				1		1								13
PA4967	*parE*		1	1	1	1	1	1	2	1					1		1	1	1	1	1	2		1	18
PA3553	*pmrF*																3	1		1	1		1		7
PA3554	*arnA*		2	4	4	5	4	4	3	2	3		3		4		6		2			2	3	4	55
PA0920	*mprF*		6	6	6	4	6	6	9		2	1	1	2	7		8	2	1	2	2	1	2	2	76
		Total SNPs	137	140	144	124	146	150	136	82	89	79	83	77	125	0	217	88	109	88	81	101	87	86	

### Virulence genes

**Figure 4:**
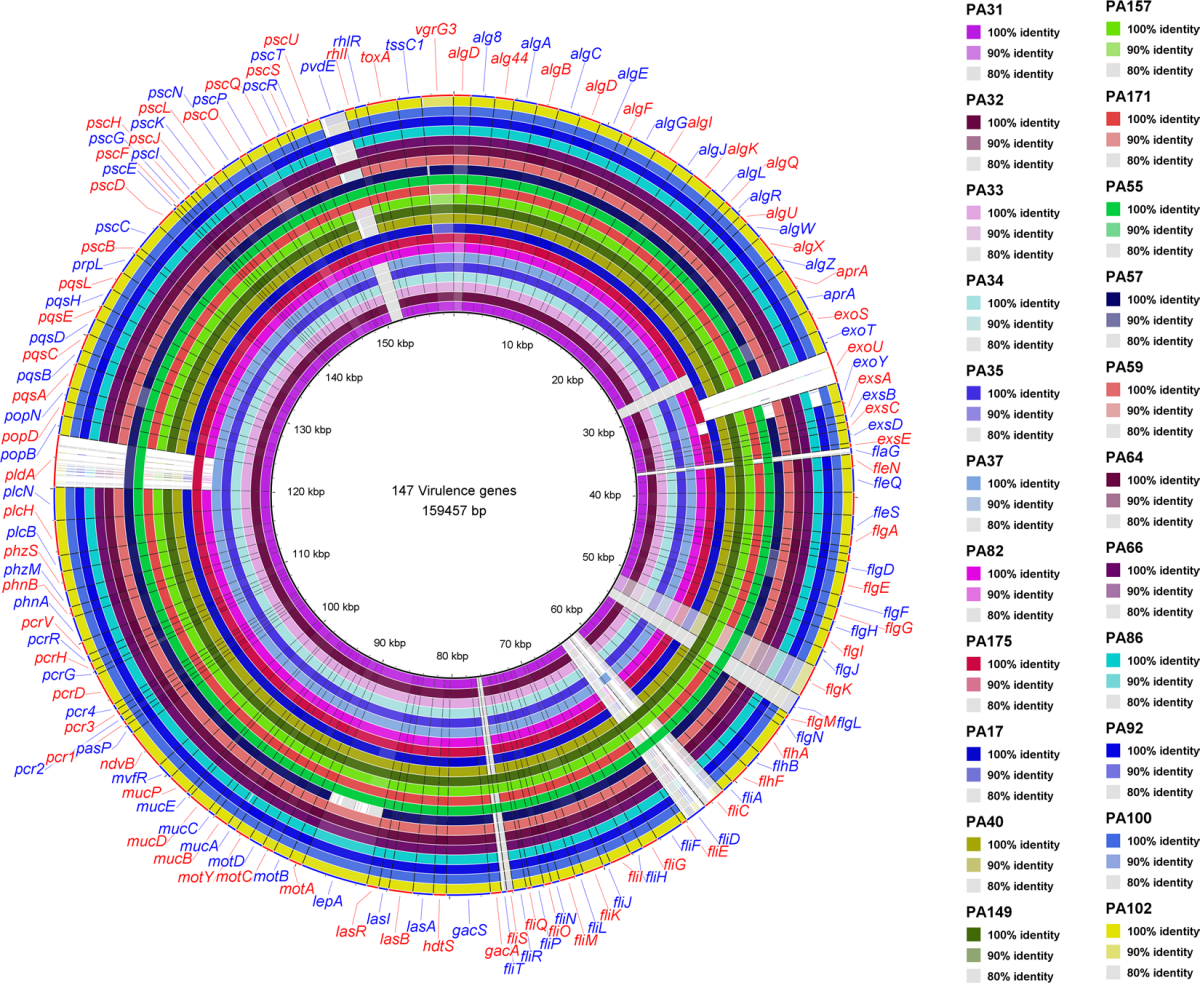
A circular representation of the genomes of studied isolates. The draft genomes of 22 strains were aligned against the 147 virulence genes curated from VFDB. Each genome is represented by a ring with different colours, which are shown in figure. Image was generated using BRIG (http://brig.sourceforge.net).

Virulence factors associated with keratitis and cystic fibrosis were selected based on the literature and published sequences in the Virulence Factor Data Base (VFDB)^52^ to examine the presence or absence of genes related to pathogenicity in the strains. A dataset of 147 virulence genes of PAO1 associated with adherence, protease production, the type IV secretion system, quorum sensing, alginate production/regulation and toxins were curated from VFDB and used in BLAST searches (Fig. 4). For the *exoU* gene, PA14 was taken as the reference because it is not present in PAO1. All instances where there was an absence of a gene were manually examined with orthologs from the most widely studied strains recommended by the PGDB^28^. Out of 147 genes, variation in virulence genes were found for 20 genes. This was most evident for a set of effector proteins (toxins) related to the type III secretion system (*exoS*, *exoT*, *exoU* and *exoY*)^53,54^. As in previous studies, *exoS* was predominantly found in CF strains (present in eight out of nine strains) and *exoU* was primarily found in eye strains (present in eight out of 13 eye isolates)^25,55–58^. Furthermore, as determined by previous studies, *exoU* and *exoS* were mutually exclusive^59^. However, neither *exoU* nor *exoS* was detected in the CF strain PA57. As the *exoU* gene is carried by genomic islands^53,60^, *exoU* possessing strains showed larger accessory genomes and cluster together in the same phylogenetic group. The *exoT* (100%) and *exoY* (86%) genes were the most prevalent secretory toxins in the strains and this result is in agreement with previous findings^61^. In a recent study, *exoY* (55%) and *exoT* (5%) were less prevalent than in the current study although the reason for these differences in distribution remains unclear^62^. One possible reason for this difference is that the study examined genes on the basis of PCR products, which may not be able to capture all different orthologs of genes.

Flagellar genes help in the establishment of infections as they can be involved in adherence to surfaces and were also widely variable between strains^63^. Seven flagellar genes (*flgK*, *flgL*, *fliC*, *flaG*, *fliD*, *fliS*, and *fliT*) clustered between PA1086 and PA1096 in PAO1 were not matched with those of 19 strains that included both eye and CF isolates. However, these genes from 19 strains showed 90% to 99% similarity with genes between PA7_4275 and PA7_4291 of PA7, orthologs of the above seven flagellar genes. There was low sequence similarity (<50%) for the above flagellar genes between PAO1 and PA7. Studies involving CF isolates have shown that the activity of the *fliC* gene (that encodes flagellin) had been either downregulated^64^ or was absent in some strains^63^. As flagella are immunogenic, the loss of flagella may be an important antiphagocytic mechanism in chronic infection isolates^65^. Although it has been shown that non-flagellated strains are defective in acute infections^65^, 85% of eye isolates in this study had altered flagellar genes that may affect flagellar function. Previous work has shown that although *fliC* contributes to invasion of *P*. *aeruginosa* in eye infections, a lack of *fliC* did not cause complete loss of invasion^66^. Further studies will need to clarify the functionality of those flagellar genes on studied strains and their role in ocular *P*. *aeruginosa* infections.

A phospholipase D gene (*pldA*), a part of the type VI secretion system of *P*. *aeruginosa* is believed to promote chronic infections^67,68^. However, *pldA* was absent from 13 isolates, seven of which were CF isolates and yet over 50% eye isolates had *pldA*. Reports on the role of *pldA* in eye infections have not been published and this should be an area of future study. Another notable variation was observed in *pvdE*, a precursor for pyoverdin synthesis, which is essential for virulence of *P*. *aeruginosa*^69,70^. Eight strains, irrespective of their source of isolation, had a PAO1 homolog of *pvdE*. Similarly, DK2 and LES homologs of *pvdE* were equally distributed in 14 strains (Table 4) suggesting that these orthologs are evenly distributed in *P*. *aeruginosa* populations. PvdE can increase invasion of *P*. *aeruginosa* by inducing expression of the *exoS*^71^. Further studies will help understand role of *pvdE* variants in pathogenesis.

**Table 4:** Distribution of *pvdE* orthologs among strains.

Strains	*pvdE* orthologs (locus tag)
PA31, PA32, PA33, PA34, PA35, PA37 and PA175	*P*. *aeruginosa* DK2 (DK2_13280)
PA82, PA17, PA171, PA175, PA55, PA57, PA64, and PA92	*P*. *aeruginosa* PAO1 (PA2397)
PA40, PA149, PA59, PA66, PA86, PA100 and PA102	*P*. *aeruginosa* LESB58 (PALES_28991)

## Conclusions

This study compared the genomic variations between Australian and Indian *P*. *aeruginosa* isolates from ocular infections. *P*. *aeruginosa* isolates from various sources showed diversity in the size of accessory genome, antibiotic resistance genes and virulence factors. We found a slightly smaller core genome than has been reported previously. Although all 22 strains were distributed throughout the global phylogeny of *P*. *aeruginosa*, certain clusters were observed in the eye isolates where five Indian eye isolates were clustered into a single clonal lineage in the group which also contains a well-studied and virulent strain PA14. Larger accessory genomes were associated with eye isolates of this group. Furthermore, the strains of this group had more SNPs in their set of 73 resistome suggesting possible positive antibiotic selection pressure. Variation in virulence factors, except for *exoU,* was not correlated with phylogeny. This study relied on draft genomes and may not be able to predict actual genomic diversity because the analysis could not ascertain the presence of the plasmids in any of the isolates. Further studies will focus on improvement of the assembly of these genomes. Overall, these findings have extended our understanding of the genomic diversity of *P*. *aeruginosa* in two different infections and information can be used to elucidate various mechanism that would help fight against virulent and drug resistant strains.

## Methods

### Bacterial strains and antibiotic susceptibility tests

Twenty two clinical isolates of *P*. *aeruginosa* from corneas of microbial keratitis and from the lungs of CF patients were selected for this study. Seven ocular isolates were obtained from a tertiary eye care centre in India (L.V. Prasad Eye Institute, Hyderabad, India), six ocular and nine CF isolates were acquired from various sources in Australia. All strains were collected from institutional repositories between 1992 and 2007 without identifiable patient data and all experiments followed the institutional guidelines, which were in place at the time (Table 5). Genome sequence data of an additional 82 *P*. *aeruginosa* strains, based on availability of complete genome sequence in Pseudomonas genome database (PGDB) version 17.2^28^ including *P*. *aeruginosa* PAO1 (reference strain) were collected from public databases and used in this study to compare results and to build phylogenetic trees (all the reference strains used in this study are listed in Supplementary Table S1). The minimum inhibitory concentrations (MICs) of ceftazidime, cefepime, aztreonam, ticarcillin, imipenem, gentamicin, levofloxacin, ciprofloxacin, moxifloxacin and polymyxin were determined by broth microdilution according to CLSI guidelines and published standard breakpoints^72–74^.

**Table 5:** List of strains used in this study.

Strains	Collection date^#^	Geographical location	Associated infections
PA31	02/10/1997	LVPEI, Hyderabad, India	Microbial Keratitis
PA32	08/10/1997	LVPEI, Hyderabad, India	Microbial Keratitis
PA33	29/08/1997	LVPEI, Hyderabad, India	Microbial Keratitis
PA34	28/08/1997	LVPEI, Hyderabad, India	Microbial Keratitis
PA35	09/08/1997	LVPEI, Hyderabad, India	Microbial Keratitis
PA37	11/07/1997	LVPEI, Hyderabad, India	Microbial Keratitis
PA82	11/05/2004	LVPEI, Hyderabad, India	Microbial Keratitis
PA17	15/09/1992	Flinders, Adelaide, Australia	Microbial Keratitis
PA40	02/02/1999	SEH, Sydney, Australia	Microbial Keratitis
PA149	04/03/2004	Flinders, Adelaide, Australia	Microbial Keratitis
PA157	29/04/2006	PAH, Brisbane, Australia	Microbial Keratitis
PA171	16/03/2006	PAH, Brisbane, Australia	Microbial Keratitis
PA175	07/10/2006	PAH, Brisbane, Australia	Microbial Keratitis
PA55	2003	RPAH, Sydney, Australia	Cystic Fibrosis
PA57	2003	RPAH, Sydney, Australia	Cystic Fibrosis
PA59	2003	RPAH, Sydney, Australia	Cystic Fibrosis
PA64	2003	RPAH, Sydney, Australia	Cystic Fibrosis
PA66	2003	RPAH, Sydney, Australia	Cystic Fibrosis
PA86	2004	RPAH, Sydney, Australia	Cystic Fibrosis
PA92	2004	RPAH, Sydney, Australia	Cystic Fibrosis
PA100	2004	RPAH, Sydney, Australia	Cystic Fibrosis
PA102	2004	RPAH, Sydney, Australia	Cystic Fibrosis

LVPEI = LV Prasad Eye Institute; Flinders = Flinders University, SEH = Sydney Eye Hospital; PAH = Princes Alexandra Hospital; RPAH = Royal Prince Alfred Hospital CF Clinic, Sydney, Australia.

^#^All cystic fibrosis isolates were obtained from Royal Prince Alfred Hospital CF Clinic, Sydney, Australia, between 2003 and 2004. Information on exact date of collection is missing in our record.

### Whole genome sequencing

Genomic DNA was extracted from overnight cultures using the DNeasy^®^ Blood and Tissue Kit (QIAGEN^®^, Germany) following the manufacturer’s instructions. The paired-end library was prepared using Nextera XT DNA library preparation kit (Illumina^®^, San Diego, CA, USA). Libraries were then sequenced on Illumina^®^ MiSeq bench top sequencer (Illumina), generating 300 bp paired-end reads. All of the libraries were multiplexed on one MiSeq run.

### Genome assembly and sequence analysis

The MiSeq sequencing resulted an average of 760,773 reads (range 632,180 to 1,193,844) per isolate. FastQC version 0.11.7 (https://www.bioinformatics.babraham.ac.uk/projects/fastqc) was used to assess the quality of raw reads, which were then quality trimmed to remove adaptor sequences using Trimmomatic version 0.36 and with the setting of minimum read length of 36 and minimum coverage of 15^75^. A *de novo* assembly was performed by SPAdes version 3.11.1^76^. with the default setting. The annotations of the assembled genomes were performed using Prokka version 1.7. using GenBank^®^ compliance flag^77^. The genome of *P*. *aeruginosa* PAO1 (RefSeq accession number NC_002516.2), which was used as the reference in this study, was re-annotated with Prokka to avoid annotation bias. Whenever necessary, the contigs of the draft genomes were reordered and/or aligned with the reference genome using MAUVE multiple-genome alignment software^78,79^. Artemis, a genome browser tool^80^, was used to concatenate the ordered contigs to get a single fragment of genomes which were used to examine insertion sequence using web tool ISsaga (http://issaga.biotoul.fr/ISsaga2/issaga_index.php), genomic islands using IslandViewer 4^81^ and prophages using PHASTER^82^ Multi locus sequence type (MLST) was determined using pubMLST database^38^ to find sequence type (ST) of each strain.

### Pan-genome and Phylogenetics

The pangenome analysis was performed using Roary version 3.12.0^83^ which uses the GFF3 files produced by Prokka. The program was run using the default settings, which uses BLASTp for all-against-all comparison with a percentage sequence identity of 95%. Core-genes were taken as the genes which were common in at least 99% of strains. The accessory genome was obtained as the genes present in the genome of each strain minus core genes. The Roary “gene_presence_absence.csv” file was further examined for unique genes using “union” and “difference” command. Parsnp version 1.2 in the Harvest Suite^84^ was used to align the genomes of 104 *P*. *aeruginosa* strains (82 complete genomes from the PGDB and 22 draft genomes from this study), followed by the construction of a maximum likelihood tree based on core genome single nucleotide polymorphisms (SNPs), excluding SNPs identified in regions that had arisen by recombination.

### Variant calling

The paired-end reads for each isolate were aligned against the genome of the *P*. *aeruginosa* PAO1 using Bowtie2 version 2.3.2^85^ following “score-min” command to avoid alignments that score less than the default minimum score threshold and with “local” flag for better score. Genomic variants were compiled using “mpileup” in SAMtools, version 1.7^86^. A minimum quality score of 50 was set to list the SNPs and Indels. The genomic variants were annotated using SnpEff version 4.3^87^ with the default options to obtain the nucleotide changes and the predicted effects at the protein level.

### Antibiotic resistance and virulence genes

Genomes were examined for the presence of acquired resistance genes using Resfinder 3.0 (Centre for Genomic Epidemiology, DTU, Denmark)^88^. Furthermore, a set of 73 genes related to antibiotic and disinfectant resistance in *P*. *aeruginosa* were selected from searches in the online databases Comprehensive Antibiotic Resistance Database (CARD) (https://card.mcmaster.ca/home)^89^ and Pseudomonas genome database (http://www.pseudomonas.com)^28^. These 73 genes were manually examined for the presence of non-synonymous SNPs to predict genotypic changes in the resistome (see Supplementary Table S3).

A dataset of 146 virulence genes of PAO1 and one virulence gene (*exoU*) of *Pseudomonas aeruginosa* UCBPP-PA14 (NC_008463.1) associated with adherence (flagella), protease production, type IV secretion system, quorum sensing, alginate production/regulation and toxins were curated from the Virulence Factor Data Base (VFDB)^90^ and used in BLAST searches to match them with the genomes of the strains studied here. BLAST Ring Image Generator (BRIG)^91^ was used to generate an image that shows presence or absence of virulence genes in multiple genomes. (List of virulence genes used is shown in Supplementary Table S4). The contigs were joined together before searching them in BRIG to avoid false matching due to fragmented genomes. The absence of a gene in this analysis was confirmed by manual BLASTn searching using orthologs from a widely-studied panel of *P*. *aeruginosa* suggested by PGDB. These include PA14, *P*. *aeruginosa* LESB58 (NC_011770.1), *P*. *aeruginosa* PA7 (NC_009656.1) and *P*. *aeruginosa* DK2 (CP003149.1).

### Nucleotide accession

The nucleotide sequences are available in the GenBank under the Bioproject accession number PRJNA431326.

## Electronic supplementary material

Supplementary Information
