# Supplementary material for: Comparative genomics of clinical strains of Pseudomonas aeruginosa strains isolated from different geographic sites
Source: Sci Rep. 2018 Oct 23;8:15668. doi: 10.1038/s41598-018-34020-7 (PMC6199293; doi:10.1038/s41598-018-34020-7)
Supplement: Supplementary file 1 — Supplementary Information [file 41598_2018_34020_MOESM1_ESM.pdf]

Supplementary information

Comparative genomics of clinical strains of *Pseudomonas aeruginosa* strains isolated from different geographic sites

Dinesh Subedi,[a#] Ajay Kumar Vijay,[a] Gurjeet Singh Kohli,[b] Scott A. Rice,[b,c,d] and Mark Willcox,[a]

[a]School of Optometry and Vision Science, University of New South Wales, Sydney, Australia, [b]The Singapore Centre for Environmental Life Sciences Engineering and [c]The School of Biological Sciences, Nanyang Technological University, Singapore, [d]The ithree Institute, The University of Technology Sydney, Sydney NSW Australia.

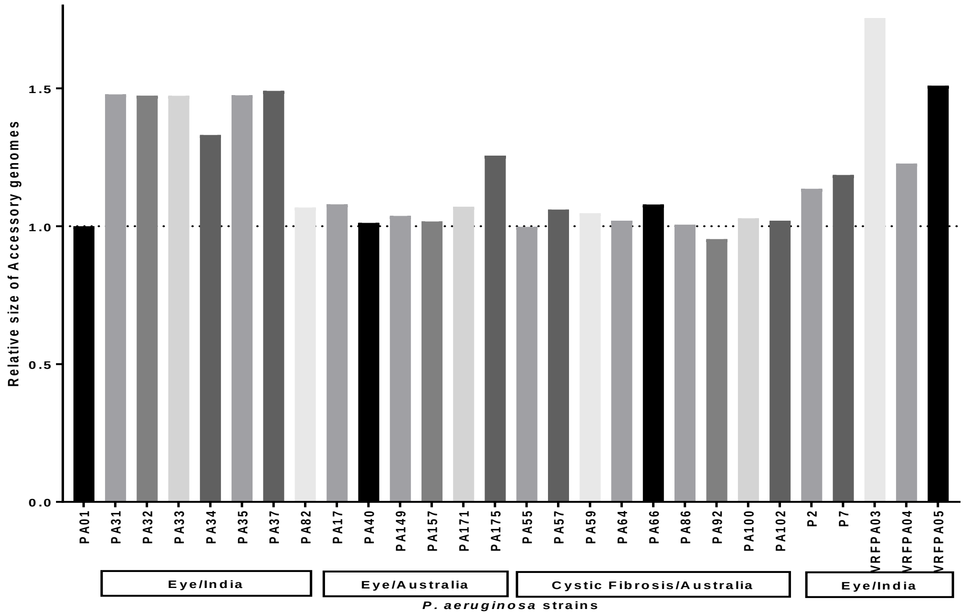

Supplementary Figure S1: Size of accessory genomes of 22 strains of this study and five published sequences of Indian *P. aeruginosa* isolates from eye infections relative to PAO1. Dotted line represents size of accessory genome of PAO1. The reference genomes Genbank® accession numbers *P. aeruginosa* VRFPA04 (CP008739.2*) P. aeruginosa* VRFPA03 (ATNK00000000.1), *P. aeruginosa* VRFPA05 (AXZJ00000000.1) *P. aeruginosa* P2-L230/9 (JMBR00000000) *P. aeruginosa* P7-L633/96 (JMBS02000000).

Supplementary Table S1: Strains of *Pseudomonas aeruginosa* used in the study downloaded from Pseudomonas genome database based on availability of complete genome

Strain	Number of Genes	GenBank Accession	Phylogeny group	Isolation Source	Core genome SNPs
**PAO1 (Reference)**	5688	NC_002516.1	1	Wound	0
**UCBPP-PA14**	5977	NC_008463.1	2	burn wound	28500
**PA7**	6369	NC_009656.1	1	non-respiratory clinical isolate	176550
**LESB58**	6028	NC_011770.1	1	sputum	13325
**VA-134**	5804	CP013245.1	1	skin wound of burn human patient	13663
**X78812**	5782	CP008872.2	1	missing	13872
**F9670**	5987	CP008873.1	1	missing	13722
**F22031**	6077	CP007399.1	1	pubic bone	13687
**NCTC10332**	5704	LN831024.1	1	not known	13685
**12-4-4(59)**	5876	CP013696.1	1	Blood culture of burn human patient	13411
**DSM50071**	5803	CP012001.1	1	missing	13681
**S86968**	6360	CP008865.2	1	missing	13657
**T38079**	6137	CP008866.2	1	missing	13658
**T52373**	5643	CP008867.1	1	missing	13708
**T63266**	5880	CP008868.1	1	missing	13731
**W16407**	6181	CP008869.2	1	missing	11626
**W36662**	6237	CP008870.2	1	missing	13698
**W45909**	6225	CP008871.2	1	missing	13364
**W60856**	6333	CP008864.2	1	missing	13232
**F23197**	5905	CP008856.2	1	missing	14694
**F30658**	6622	CP008857.1	1	missing	14147
**H5708**	5804	CP008859.2	1	missing	14287
**H27930**	5964	CP008860.2	1	missing	13418
**H47921**	6249	CP008861.1	1	missing	18295
**M1608**	5887	CP008862.2	2	missing	27703
**M37351**	6322	CP008863.1	2	missing	27702
**USDA-ARS-USMARC-41639**	5814	CP013989.1	1	nasopharynx	13740
**NCGM1984**	6399	AP014646.1	2	catheter	27399
**NCGM1900**	6354	AP014622.1	2	catheter	27399
**FRD1**	6179	CP010555.1	1	sputum	14145
**Carb01 63**	7070	CP011317.1	1	environment	15254
**DHS01**	6608	CP013993.1	1	nose	13536
**NCGM257**	6628	AP014651.1	2	Midstream urine	28392
**8380**	6086	AP014839.2	1	environment	13960
**IOMTU 133**	6276	AP017302.1	2	Urinary catheter	28311
**PACS2**	5989	AAQW01000001.1	1	missing	14351
**F9676**	5827	CP012066.1	1	environment	13151
**PA1088**	6196	CP015001.1	1	urine	13860
**213BR**	6169	AFXK01000001.1	1	missing	13866
**NHmuc**	5795	CP013479.1	1	missing	13484
**19BR**	6189	AFXJ01000001.1	1	missing	13867
**PA1RG**	5994	CP012679.1	1	hospital sewage	15635
**F63912**	6149	CP008873.1	1	missing	13747
**N17-1**	5867	CP014948.1	1	soil	13451
**PAER4_119**	6083	CP013113.1	1	Lindberg collection	13806
**ATCC 27853**	6312	CP015117.1	1	missing	13649
**BAMCPA07-48**	6438	CP015377.1	2	combat injury wound	27990
**PA121617**	6303	CP016215.1	1	sputum	13407
**PA_154197**	6041	CP017306.1	1	missing	12908
**PA_D1**	6069	CP012585.1	2	sputum	27830
**PA_D2**	6066	CP012578.1	2	sputum	27833
**PA_D9**	6065	CP012580.1	2	sputum	27830
**PA_D16**	6086	CP012581.1	2	sputum	27831
**PA_D22**	6091	CP012583.1	2	sputum	27831
**SCVJan**	5794	CP013478.1	1	murine model	13483
**PA_D25**	6080	CP012584.1	2	sputum	27843
**PA_D5**	6087	CP012579.1	2	sputum	27830
**PA_D21**	6063	CP012582.1	2	sputum	27834
**ATCC 15692**	5744	CP017149.1	1	Infected wound	14
**FA-HZ1**	6147	CP017353.1	1	wastewater	13349
**M18**	5770	CP002496.1	1	missing	12888
**NCGM2.S1**	6274	AP012280.1	2	missing	27404
**PA14Or_reads**	5971	LT608330.1	2	missing	28430
**PcyII-10**	5847	LT673656.1	1	missing	13188
**DK2**	5959	CP003149.1	1	sputum	14269
**B136-33**	5904	CP004061.1	2	diarrhea	27981
**RP73**	5864	CP006245.1	1	missing	13795

**Strain**	**Number of Genes**	**GenBank Accession**	**Phylogeny group**	**Isolation Source**	**Core genome SNPs**
**YL84**	5908	CP007147.1	1	compost	13980
**DN1**	6626	CP017099.1	2	soil	27667
**MTB-1**	6186	CP006853.1	2	t-HCH contaminated soil	28100
**SCV20265**	6380	CP006931.1	1	lung	13980
**VRFPA04**	6308	CP008739.2	2	corneal button	27727
**SCVFeb**	5794	CP013477.1	1	murine model	13483
**LES431**	6091	CP006937.1	1	missing	13335
**PA11803**	6565	CP015003.1	1	bloodstream	13874
**PAO1_Orsay**	5757	LN871187.1	1	missing	13
**PA8281**	6408	CP015002.1	1	tracheal aspirate	13873
**PA1R**	5689	CP004055.1	1	missing	15639
**PA1**	5987	CP004054.2	1	missing	15636
**PA7790**	6577	CP015000.1	1	tracheal aspirate	13875
**B10W**	6317	CP017969.1	2	wastewater	27524
**Ocean_1155**	6481	CP022526.1	2	Ocean	27659

Supplementary Table S2: Single Nucleotide Polymorphisms (SNPs) observed in core genome of 22 strains of *P. aeruginosa*

P. aeruginosa **strains**	**Core genome SNPs (reference to PAO1)**
**PA31**	27547
**PA32**	27607
**PA33**	27607
**PA34**	28502
**PA35**	27608
**PA37**	27609
**PA82**	27990
**PA17**	13484
**PA40**	14442
**PA149**	13898
**PA157**	14248
**PA171**	13345
**PA175**	27411
**PA55**	14
**PA57**	46459
**PA59**	13844
**PA64**	14133
**PA66**	13856
**PA86**	13851
**PA92**	14129
**PA100**	15067
**PA102**	13381

Supplementary Table S3: List of resistome and associated functions

Locus tag (PAO1)	Gene	Product/Function
**PA0004**	*gyrB*	DNA gyrase subunit B
**PA0156**	*triA*	Resistance-Nodulation-Cell Division (RND) triclosan efflux membrane fusion protein, TriA
**PA0157**	*triB*	Resistance-Nodulation-Cell Division (RND) triclosan efflux membrane fusion protein, TriB
**PA0158**	*triC*	Resistance-Nodulation-Cell Division (RND) triclosan efflux transporter, TriC
**PA0424**	*mexR*	multidrug resistance operon repressor
**PA0425**	*mexA*	Resistance-Nodulation-Cell Division (RND) multidrug efflux membrane fusion protein MexA precursor
**PA0426**	*mexB*	Resistance-Nodulation-Cell Division (RND) multidrug efflux transporter MexB
**PA0427**	*oprM*	Major intrinsic multiple antibiotic resistance efflux outer membrane protein OprM precursor
**PA0706**	*catB7*	chloramphenicol acetyltransferase
**PA0903**	*alaS*	alanyl-tRNA synthetase
**PA0920**	*mprF*	alanyl-phosphatidylglycerol synthase
**PA1129**	*fosA*	fosfomycin resistance protein, FosA
**PA1236**	*farB*	probable major facilitator superfamily (MFS) transporter
**PA1282**	*lrfA*	probable major facilitator superfamily (MFS) transporter
**PA1316**	*lrfA*	probable major facilitator superfamily (MFS) transporter
**PA1435**	*mexM*	probable Resistance-Nodulation-Cell Division (RND) efflux membrane fusion protein precursor
**PA1436**	*mdtC*	probable Resistance-Nodulation-Cell Division (RND) efflux transporter
**PA1754**	*cysB*	transcriptional regulator CysB
**PA1972**	*pmrC*	conserved hypothetical protein
**PA2018**	*mexY*	Resistance-Nodulation-Cell Division (RND) multidrug efflux transporter MexY
**PA2019**	*mexX*	Resistance-Nodulation-Cell Division (RND) multidrug efflux membrane fusion protein MexX precursor
**PA2389**	*macA*	Membrane fusion protein that forms an antibiotic efflux complex with MacB and TolC.
**PA2390**	*macB*	ATP-binding cassette (ABC) transporter
**PA2391**	*opmQ*	probable outer membrane protein precursor
**PA2491**	*mexS*	suppressor of MexT
**PA2493**	*mexE*	Resistance-Nodulation-Cell Division (RND) multidrug efflux membrane fusion protein MexE precursor
**PA2494**	*mexF*	Resistance-Nodulation-Cell Division (RND) multidrug efflux transporter MexF
**PA2495**	*oprN*	Multidrug efflux outer membrane protein OprN precursor
**PA2525**	*adeC*	outer membrane factor of the AdeABC multidrug efflux complex

Locus tag (PAO1)	Gene	Product/Function
**PA2526**	*muxC*	transporter that forms a heteromultimer complex with MdtB to form a multidrug transporter
**PA2527**	*muxB*	transporter that forms a heteromultimer complex with MdtB to form a multidrug transporter
**PA2837**	*opmA*	probable outer membrane protein precursor
**PA3002**	*mfd*	transcription-repair coupling protein Mfd
**PA3019**	*taeA*	probable ATP-binding component of ABC transporter
**PA3137**	*farB*	probable major facilitator superfamily (MFS) transporter
**PA3168**	*gyrA*	DNA gyrase subunit A
**PA3521**	*opmE*	outer membrane factor protein that is part of the multidrug efflux pump MexPQ-OpmE
**PA3522**	*mexQ*	inner membrane transporter of the AdeFGH multidrug efflux complex
**PA3523**	*mexP*	membrane fusion protein of the MexPQ-OpmE multidrug efflux complex
**PA3553**	*PmrF*	required for the synthesis and transfer of 4-amino-4-deoxy-L-arabinose (Ara4N) to Lipid A for resistance to polymyxin
**PA3554**	*arnA*	modifies lipid A with 4-amino-4-deoxy-L-arabinose (Ara4N)
**PA3574**	*nalD*	repressor of MexAB-OprM
**PA3676**	*mexK*	inner membrane resistance-nodulation-cell division (RND) transporter in the MexJK multidrug efflux protein
**PA3677**	*mexJ*	membrane fusion protein of the MexJK multidrug efflux protein
**PA3678**	*mexL*	specific repressor of mexJK transcription and autoregulates its own expression
**PA3894**	*adeC*	probable outer membrane protein precursor
**PA3946**	*rosC*	two-component sensor RocS1
**PA4109**	*ampR*	Transcriptional Regulator
**PA4110**	*ampC*	beta-lactamase precursor
**PA4119**	*APH(3')-IIb*	aminoglycoside 3'-phosphotransferase type IIb
**PA4205**	*mexG*	hypothetical protein
**PA4206**	*mexH*	probable Resistance-Nodulation-Cell Division (RND) efflux membrane fusion protein precursor
**PA4207**	*mexI*	probable Resistance-Nodulation-Cell Division (RND) efflux transporter
**PA4208**	*opmD*	probable outer membrane protein precursor
**PA4265**	*tufA*	elongation factor Tu
**PA4277**	*tufB*	elongation factor Tu
**PA4374**	*mexV*	Resistance-Nodulation-Cell Division (RND) multidrug efflux membrane fusion protein MexV
**PA4375**	*mexW*	Resistance-Nodulation-Cell Division (RND) multidrug efflux transporter MexW
**PA4560**	*ileS*	isoleucyl-tRNA synthetase
**PA4595**	*yjjk*	probable ATP-binding component of ABC transporter
**PA4597**	*oprJ*	Multidrug efflux outer membrane protein OprJ precursor

**Locus tag (PAO1)**	**Gene**	**Product/Function**
**PA4598**	*mexD*	Resistance-Nodulation-Cell Division (RND) multidrug efflux transporter MexD
**PA4599**	*mexC*	Resistance-Nodulation-Cell Division (RND) multidrug efflux membrane fusion protein MexC precursor
**PA4600**	*nfxB*	transcriptional regulator NfxB
**PA4964**	*parC*	topoisomerase IV subunit A
**PA4967**	*parE*	topoisomerase IV subunit B
**PA4974**	*opmH*	probable outer membrane protein precursor
**PA4990**	*emrE*	SMR multidrug efflux transporter
**PA4997**	*msbA*	transport protein MsbA
**PA5158**	*adeC*	probable outer membrane protein precursor
**PA5160**	*farB*	drug efflux transporter
**PA5514**	*OXA-50*	probable beta-lactamase
**PA5518**	*rosB*	probable potassium efflux transporter

Supplementary Table S4: List of virulence genes used in the study

Virulence genes	Functions
alg44	Type 4 fimbrial precursor
alg8	Alginate biosynthesis protein
algA	Alginate-c5-mannuronan-epimerase
algB	Phosphomannose isomerase/guanosine 5'-diphospho-D-mannose pyrophosphorylase
algC	Two-component response regulator
algD	Phosphomannomutase
algE	GDP-mannose 6-dehydrogenase
algF	Alginate biosynthetic protein AlgK precursor
algG	Alginate o-acetyltransferase
algI	Outer membrane protein
algJ	Alginate o-acetyltransferase
algK	Alginate o-acetyltransferase
algL	Alginate biosynthesis protein
algP/algR3	Poly(beta-d-mannuronate) lyase precursor
algQ	Alginate regulatory protein
algR	Alginate regulatory protein
algU	Alginate biosynthesis regulatory protein
algW	Alginate biosynthesis protein
algX	AlgW protein [Alginate regulation (CVF523)]
algZ	Alginate biosynthesis protein
aprA	Sigma factor
aprA	Sigma factor
exoS	Hypothetical protein
exoT	Type III secretion system effector
exoU*	Type III secretion system effector
exoY	Type III secretion system effector
exsA	Type III secretion system effector
exsB	Type III secretion system regulatory protein
exsC	Type III secretion system piolitin
exsD	Type III secretion system regulatory protein
exsE	Type III secretion system regulatory protein
fleI/flag	FimX [type IV pili (AI097)]
fleN	Flagellar protein FlaG [Deoxyhexose linking sugar]
fleP	Flagellar synthesis regulator
fleQ	Flagellar protein
fleR	Transcriptional regulatore
fleS	Two-component response regulator
flgA	Two-component sensor [Deoxyhexose linking sugar, 209 linking sugar, island]
flgB	Flagellar basal body P-ring biosynthesis protein linking Da
flgC	Flagellar basal body rod protein

Virulence genes	Functions
flgD	Flagellar basal-body rod protein
flgE	Flagellar basal-body rod modification protein
flgF	Flagellar hook protein
flgG	Flagellar basal-body rod protein
flgH	Flagellar basal-body rod protein
flgI	Flagellar L-ring protein precursor
flgJ	Flagellar P-ring protein precursor
flgK	Flagellar rod assembly protein/muramidase
flgL	Flagellar hook-associated protein 1
flgM	Flagellar hook-associated protein 3
flgN	Negative regulator of flagellin synthesis
flhA	Flagella synthesis protein
flhB	Flagellar biosynthesis protein
flhF	Flagellar biosynthetic protein
fliA	Flagellar biosynthesis protein
fliC	Flagellar biosynthesis sigma factor
fliD	B-type flagellin
fliE	Flagellar capping protein
fliF	Flagellar hook-basal body complex protein
fliG	Flagellar M-ring protein
fliH	Flagellar motor switch protein G
fliI	Flagellar assembly protein H
fliJ	Flagellum-specific ATP synthase
fliK	Flagellar protein
fliL	Flagellar hook-length control protein
fliM	Flagellar basal body protein
fliN	Flagellar motor switch protein
fliO	Flagellar motor switch protein
fliP	Flagellar protein
fliQ	Flagellar biosynthetic protein
fliR	Flagellar biosynthetic protein
fliS	Flagellar biosynthetic protein
fliT	Flagellar biosynthetic protein
gacA	Response regulator
gacS	Sensor/response regulator hybrid
hdtS	Hypothetical protein
lasA	Hypothetical protein
lasB	LasA protease precursor
lasI	Elastase
lasR	Autoinducer synthesis protein
lepA	Pseudomonas aeruginosa -derived large extracellular protease

Virulence genes	Functions
motA	MbtH-like protein from the pyoverdine cluster
motB	Flagellar motor protein
motC	Flagellar motor protein
motD	Flagellar motor protein
motY	Flagellar motor protein
mucA	Probable outer membrane protein precursor
mucB	Alkaline metalloproteinase precursor
mucC	Anti-sigma factor MucA, inhibitor of alg gene activity
mucD	Negative regulator for alginate biosynthesis
mucE	Serine protease MucD precursor
mucP	Small envelope protein
mvfR	Transcriptional regulator
ndVB	Required for the synthesis of periplasmic glucans, and involved in biofilm-specific resistance to a subset of antibiotics
pasP	Small protease
pcr1	Transcriptional regulator
pcr2	Type III secretion system protein
pcr3	Type III secretion system protein
pcr4	Type III secretion system protein
pcrD	Type III secretion system protein
pcrG	Type III secretion system protein
pcrH	Type III secretion system cytoplasmic regulator
pcrR	Type III secretion system regulatory protein
pcrV	Type III secretion system regulatory protein
phnA	Anthranilate synthase component I
phnB	Anthranilate synthase component II
phzM	Phenazine-modifying enzyme
phzS	Phenazine-specific methyltransferase
plcB	Phospholipase C,
plcH	Hemolytic phospholipase C precursor
plcN	Non-hemolytic phospholipase C precursor
pldA	Phospholipase D
popB	Type III secretion system hydrophobic translocator, pore
popD	Type III secretion system hydrophobic translocator, pore
popN	Type III secretion system outer membrane protein
pqsA	Pseudomonas quinolone signal response
pqsB	Pseudomonas quinolone signal response
pqsC	Quinolone signal biosynthesis
pqsD	Quinolone signal biosynthesis
pqsE	Pseudomonas quinolone signal response
pqsH	FAD-dependent monooxygenase
pqsL	Putative monooxygenase

Virulence genes	Functions
pscB	Type III secretion system protein
pscC	Type III secretion system secretin
pscD	Type III secretion system basal body protein
pscE	Type III secretion system cochaperone
pscF	Type III secretion system needle filament protein
pscG	Type III secretion system chaperone
pscH	Type III secretion system protein
pscI	Type III secretion system inner rod protein
pscJ	Type III secretion system inner MS ring IV
pscK	Type III secretion system protein
pscL	Type III secretion system protein
pscN	Type III secretion system ATPase
pscO	Type III secretion system protein
pscP	Type III secretion system protein
pscQ	Type III secretion system protein
pscR	Type III secretion system protein
pscS	Type III secretion system protein
pscT	Type III secretion system protein
pscU	Translocation protein in type III secretion
pvdE	Pyoverdine biosynthesis protein
rhlI	Autoinducer synthesis protein
rhlR	Transcriptional regulator
toxA	Exotoxin A precursor
tssC1	Type VI secretion protein
vgrG3	Hypothetical protein

*All the genes are from *P. aeruginosa* PAO1 except for *exoU* for which *P. aeruginosa* UCBPP-PA14 is taken as reference.

Supplementary Table S5: Multi-locus sequence typing (MLST) allelic profiles and sequence type of *P. aeruginosa* strains

***P. aeruginosa* strain**	**Strain group**	**Allelic type**							**Sequence type (ST)**
		acsA	*aroE*	*guaA*	*mutL*	*nuoD*	*ppsA*	*trpE*	
**PA17**	Eye/Australia	16	5	11	1	2	7	2	New
**PA31**	Eye/India	13	4	5	5	12	7	15	308
**PA32**	Eye/India	13	4	5	5	12	7	15	308
**PA33**	Eye/India	13	4	5	5	12	7	15	308
**PA34**	Eye/India	32	8	5	3	5	6	26	1284
**PA35**	Eye/India	13	4	5	5	12	7	15	308
**PA37**	Eye/India	13	4	5	5	12	7	15	308
**PA40**	Eye/Australia	15	5	36	72	1	42	19	New
**PA55**	Cystic fibrosis/Australia	7	5	12	3	4	1	7	549
**PA57**	Cystic fibrosis/Australia	111	30	31	26	48	59	32	New
**PA59**	Cystic fibrosis/Australia	11/151	84	11	3	4	4	7	New (59)
**PA64**	Cystic fibrosis/Australia	28	5	11	5	4	4	7	775
**PA66**	Cystic fibrosis/Australia	11/151	84	11	3	4	4	7	New (59)
**PA82**	Eye/India	32	5	61	13	1	6	54	1027
**PA86**	Cystic fibrosis/Australia	11/151	84	11	3	4	4	7	New (59)
**PA92**	Cystic fibrosis/Australia	28	5	11	5	4	4	7	775
**PA100**	Cystic fibrosis/Australia	116	5	6	5	3	12	68	483
**PA102**	Cystic fibrosis/Australia	28	24	91	5	4	4	14	1717
**PA149**	Eye/Australia	28	New	12	3	4	12	3	New
**PA157**	Eye/Australia	17	5	11	18	4	10	3	386
**PA171**	Eye/Australia	17	5	11	5	4	4	2	471
**PA175**	Eye/Australia	13	8	9	3	1	17	15	309

(59) new but like PA59
